# Stiffness and Atomic-Scale Friction in Superlubricant MoS_2_ Bilayers

**DOI:** 10.1021/acs.jpclett.3c01066

**Published:** 2023-06-26

**Authors:** Rui Dong, Alessandro Lunghi, Stefano Sanvito

**Affiliations:** School of Physics, AMBER and CRANN Institute, Trinity College, Dublin 2, Ireland

## Abstract

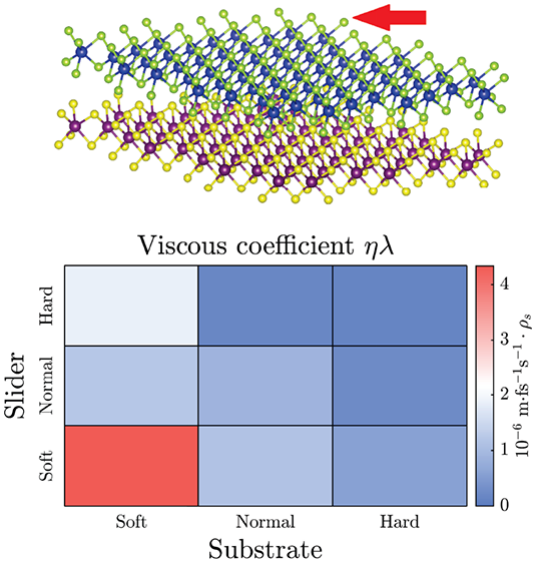

Molecular dynamics simulations, performed with chemically accurate *ab initio* machine-learning force fields, are used to demonstrate that layer stiffness has profound effects on the superlubricant state of two-dimensional van der Waals heterostructures. We engineer bilayers of different rigidity but identical interlayer sliding energy surface and show that a 2-fold increase in the intralayer stiffness reduces the friction by a factor of ∼6. Two sliding regimes as a function of the sliding velocity are found. At a low velocity, the heat generated by the motion is efficiently exchanged between the layers and the friction is independent of the layer order. In contrast, at a high velocity, the friction heat flux cannot be exchanged fast enough and a buildup of significant temperature gradients between the layers is observed. In this situation, the temperature profile depends on whether the slider is softer than the substrate.

When the lateral forces between two sliding surfaces vanish or become extremely small, the surfaces are said to be superlubricant,^1^ a state often defined by a friction coefficient of <0.01. Structural superlubricity^2^ is a particular superlubricant situation that emerges between dry and flat surfaces with incommensurate lattices. This structural peculiarity drastically suppresses the corrugation of the interlayer sliding energy surface (ISES), so that the relative motion can take place with very limited energy dissipation. The ideal conditions for structural superlubricity are found when (i) the two surfaces are extremely rigid, so that elastic deformation is prevented over long length scales, and (ii) the surface-to-surface interaction is weak.^3^

Two-dimensional (2D) materials, such as graphene, h-BN, and transition metal dichalcogenides, are characterized by strong in-plane covalent bonds and weak interlayer van der Waals interaction. Thus, vertically stacked 2D heterostructures appear as an ideal materials platform for structural superlubricity. 2D compounds have been used as solid-state lubricants for many years,^4,5^ although only recent advances in atomic force microscopy have given us a thorough microscopic understanding of the superlubricity phenomenon.^6^ One can now find experimental demonstrations of structural superlubricity in graphene,^7−10^ MoS_2_,^11−13^ and heterogeneous structures.^14−16^

Among the possible theoretical strategies for studying microscopic tribology,^17^ the “quasi-static” approach has enjoyed significant popularity. This computes the frictional forces from the gradient of the ISES,^18^ which in turn is obtained with *ab initio* methods.^19^ A variation of the same approach monitors the energy and forces during the movement of a “slider” over a “substrate”, when the internal degrees of freedom of the layers are either kept frozen^20,21^ or allowed to relax.^22^ This is a good solution when looking at effects involving extended defects, requiring large simulation cells.^23^ In general, quasi-static simulations work well when the ISES is deep and the friction is dominated by the slider center of mass (CM) scattering. However, in a superlubricant situation, the external forces dissipate into the internal atomic motion, a process that requires full molecular dynamics (MD). Then, the most typical setup consists of attaching the slider to a moving support through harmonic springs and in monitoring the spring forces over the MD trajectories, typically obtained with empirical force fields.^24−26^

Here we use highly accurate machine-learning force fields together with MD simulations without external driving forces to answer a simple but crucial question: does the stiffness of the sliding layer affect the friction of a superlubricant system? We find that indeed this is the case, a 2-fold increase in the intralayer stiffness reduces the friction by a factor of ∼6. Most importantly, the stiffness mismatch between the slider and substrate determines the thermal coupling between layers and the heat dispersion dynamics, resulting in different friction regimes at different sliding velocities.

Our simulations are for MoS_2_ bilayers. The in-plane covalent forces are described by a spectral neighbor analysis potential (SNAP),^27^ generated with a procedure described previously.^28^ In brief, we construct a data set comprising 3 × 3 supercells of the MoS_2_ monolayer primitive cell, in which both the atomic positions and the lattice vectors are distorted from the equilibrium geometry. The total energy of such configurations is computed by density functional theory (DFT) at the level of the Perdew–Burke–Ernzerhof generalized gradient approximation.^29^ The energy is then written as a sum of atomic energies, where the local atomic environment is expressed over four-dimensional bispectral components and fitted by ridge regression.^27^ The accuracy of the fit is tested against MD, and eventually, new configurations are included in the training set. This delivers us a total energy error of 1.0 meV/atom, namely an energy surface almost identical to that obtained from DFT. One can appreciate the accuracy of the SNAP from the energy parity plot and by comparing the phonon band structures of monolayer MoS_2_ obtained with both DFT and SNAP (see Figures S1 and S2). In this case, the SNAP error on the phonon frequencies is <0.25 THz.

In addition, we generate two more SNAPs, obtained by rescaling the DFT total energies by a factor of either 2 or ^1^/_2_ (the total energy is measured from that of the equilibrium configuration). The resulting SNAPs all have the same energy minimum, namely the MoS_2_ equilibrium geometry, but simulate materials with different stiffnesses (*k*). Layers described by these three SNAPs are denoted as normal, N (stiffness coefficient *k* = 8.73 meV/Å^2^), soft, S (*k* = 4.37 meV/Å^2^), and hard, H (*k* = 17.46 meV/Å^2^). Crucially, any bilayer constructed from these SNAPs has the same ISES. This allows us to perform dynamic simulations of friction in real materials with DFT accuracy and to isolate the effect of the in-plane stiffness on the superlubricity from the corrugation of the ISES. A Lennard-Jones (L-J) potential, with *C*_6_ coefficients extracted from van der Waals DFT calculations,^30^ describes the interlayer interaction. It is worth noting that more sophisticated interlayer potentials have been developed on the basis of the registry index and many-body dispersion.^41−43^ In the case of graphene and h-BN, the newly developed potentials accurately predict the ISES corrugation and have a subversive advantage over simple L-J potential. Studies also show that many-body vdW effects may become more prominent for non-equilibrium geometries.^44^ In this work, the simple L-J potential returns a bilayer binding energy of 32.1 eV/Å^2^ and an ISES corrugation of 16.3 meV/Å^2^ for the 2H order. The DFT values are 35.7 eV/Å^2^ and 23.3 meV/Å^2^, respectively. Similar results are obtained for other bilayer polymorphs. Therefore, a L-J potential should be enough to deliver the physics studied in this work, namely, to identify the role of the stiffness of a material in superlubricity.

Our elemental structure is a  cell, in which the superlubricant state is obtained by twisting by 21.8° the two layers from the 2H configuration. This is the smallest superlubricant strain-free cell possible,^31^ returning a ISES corrugation of <0.1 meV/Å^2^. Such low corrugation ensures that no interlayer locking and stick-slip motion exist in the soft layers. MD simulations are then conducted over a 6 × 6 supercell of the  cell with periodic boundary conditions. The substrate layer is kept fixed by removing the linear momentum of its CM at every MD step, while the sliding layer is set in motion with an initial velocity *v*_0_. In this way, our dynamical simulations go beyond the widely used 2D Frenkel–Kontorova model,^32,33^ where an atomic lattice held together by harmonic forces slides over a rigid 2D potential, and we can capture energy transfer and temperature equilibration between the layers. The initial velocity is set to the rather high value of *v*_0_ = 800 m/s (see Figure 1; a combustion-engine piston moves at ∼25 m/s), giving us a velocity range large enough to converge well the friction–velocity curve. Note that our very flat ISES makes the slider perform Brownian motion at a rate of ∼30 m/s in equilibrium at room temperature.

**Figure 1:**
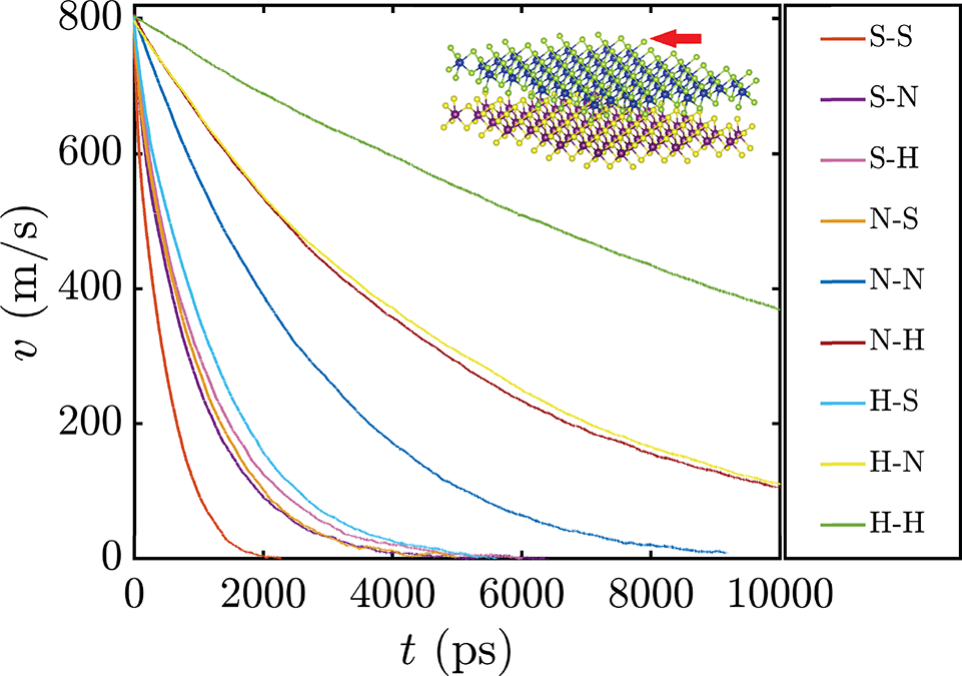
Time evolution of the velocity of the slider CM for the nine bilayer types investigated. Bilayer types are denoted as α-β, with α (β) defining the slider (substrate) and α and β = S (soft), N (normal), and H (hard). The inset shows a ball-and-stick representation of the system used in the MD simulations, where a slider MoS_2_ monolayer (top) moves above a MoS_2_ substrate (bottom). Color code: Mo, blue/purple; S, green/yellow.

The system is equilibrated at 300 K using a Nosé-Hoover thermostat^37^ for 1 ns, and then snapshots are taken every 0.5 ns and used as the starting point for the sliding. With this setup, the slider moves freely on the substrate, while its CM velocity is measured. Importantly, there is no constraint on any part of the slider, a fact that ensures that the internal atomic vibrations are not to be altered. During the sliding, the temperature of the substrate is thermostated at 300 K, but the temperature of the slider is not. This mimics experiments in a vacuum, where the slider can only exchange energy with the substrate. Bilayer types are denoted as α-β (α and β = S, N, and H) with α (β) defining the slider (substrate). For each configuration, multiple MD runs are carried out to reduce the noise, and the analysis is performed over the averaged trajectories.

Figure 1 shows the time evolution of the slider CM velocity for the nine possible bilayers. Clearly, the layer stiffness has a significant effect on the friction, because it takes only 2 ns to stop the soft MoS_2_ bilayer (from *v*_0_ = 800 m/s), while the normal and hard MoS_2_ bilayers take ∼10 and ∼40 ns, respectively. When monolayers of different stiffnesses are combined, the general trend is maintained with the harder combinations preserving the motion for longer times. An anomaly appears for the combination with the largest stiffness mismatch between the layers, because the curves for H-S (a hard layer sliding on a soft one) and S-H (a soft layer sliding on a hard one) are not identical. We will return to this point.

The acceleration–velocity curve, *a*(*v*), is obtained by the finite-difference differentiation of *v*(*t*) over 5 m/s steps. Then, the frictional force per unit area can be computed as *f*(*v*) = ρ_s_*a*(*v*), with a ρ_s_ of 18.84 a.u./Å^2^ being the 2D density of the MoS_2_ monolayer, and it is presented in Figure 2a for the soft (S-S), normal (N-N), and hard (H-H) bilayers.

**Figure 2:**
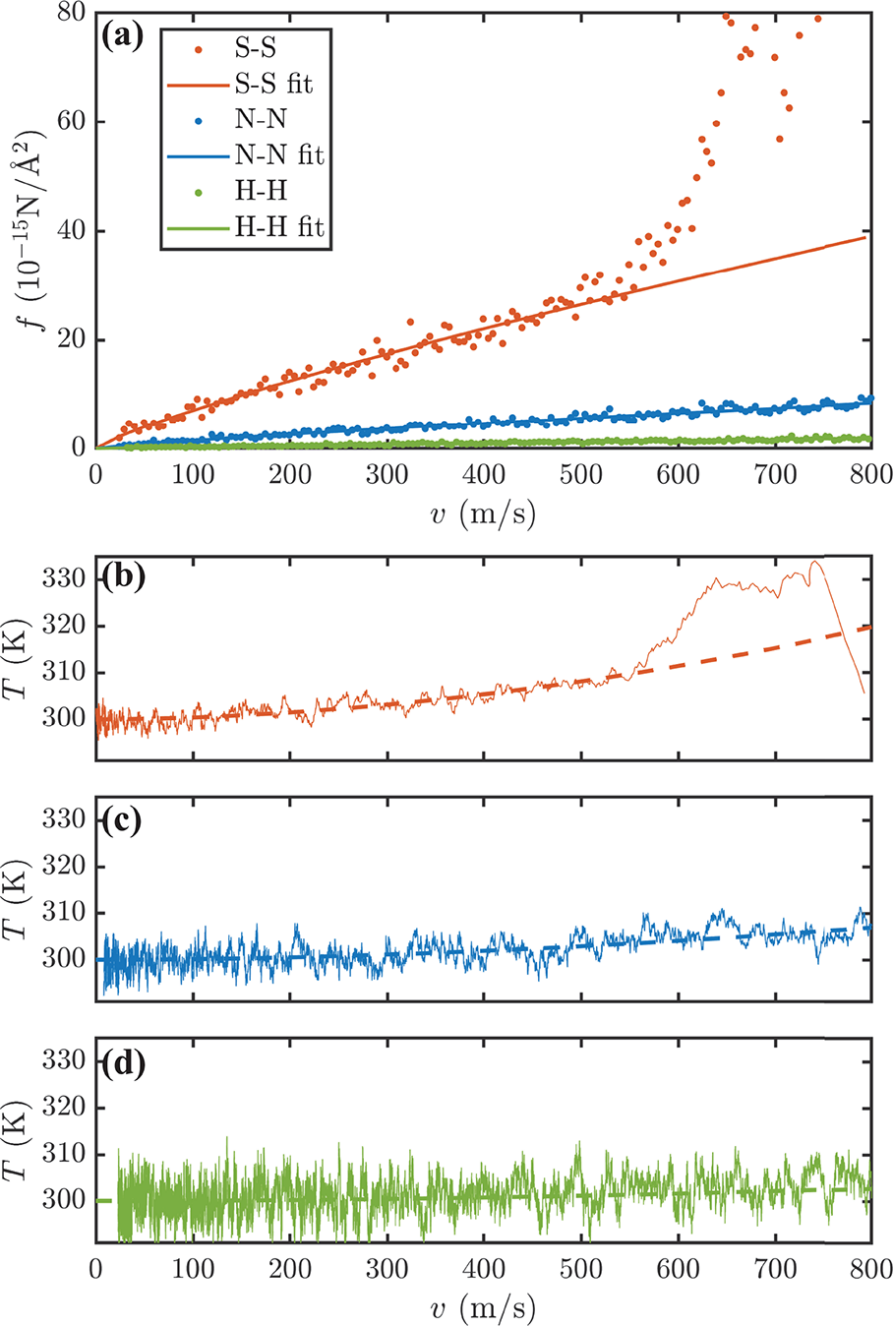
(a) Friction force, *f*, as a function of the velocity for S-S, N-N, and H-H MoS_2_ homobilayers, where the circles are for the MD data and the lines are a fit to eq 1. (b–d) Slider temperature as a function of velocity for S-S, N-N, and H-H, respectively. In panels b–d, the solid lines are from MD while the dashed ones are calculated by mean field according to eq 2.

We empirically find that the frictional force can be accurately fitted to a general expression
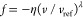
1where *v*_ref_ = 1 m/s is a reference velocity, η is the viscous coefficient, and λ is a parameter. Thus, in the velocity range explored here, *f* remains between the Stokes (*f* ∝ *v*) and Coulomb (*f* = constant) limits. This means that even in the superlubricant regime the bilayers are away from equilibrium, resulting in λ ≤ 1.^34^ Note that a sublinear dependence of the friction on the velocity is predicted by the Prandtl–Tomlinson model for stick-slip motion at a low velocity, with a crossover to constant friction in the high-*v* limit. This has also been verified by comparing experiments with MD simulations.^35,36^ However, here the situation is different, because the motion is not activated, the speeds are much higher, and the system is deep into the superlubricity limit (the friction computed here is ∼3 orders of magnitude lower than that measured in refs (35) and (36)). We notice that for the S-S system *f* increases dramatically for *v* > 550 m/s, when also the slider internal temperature steeply increases (see Figure 2b), an effect not found for the other curves up to 800 m/s. We then decide to fit the *f*(*v*) profile to eq 1 only up to a maximum velocity (*v*_max_) of ∼550 m/s, and the corresponding results are listed in Table 1.

**Table 1:** Parameters for Fitting the *f*(*v*) Curve to eq 1^a^

	η	λ	*ηλ*	*v*_max_	*R*	*G*
S-S	5.29	0.82	4.34	500	0.95	7.23
S-N	1.17	0.98	1.15	550	0.94	1.56
S-H	0.51	1.09	0.56	600	0.94	1.01
N-S	1.28	0.95	1.21	600	0.92	1.66
N-N	1.02	0.84	0.86	750	0.95	4.26
N-H	0.26	0.96	0.25	750	0.92	0.51
H-S	1.97	0.94	1.85	600	0.94	1.05
H-N	0.20	1.00	0.20	750	0.93	0.50
H-H	0.14	0.90	0.13	800	0.91	2.39

a *η* is the viscous coefficient in 10^–6^ m fs^–1^ s^–1^ × *ρ*_s_ (*ρ*_s_ = 18.84 a.u./Å^2^). *λ* is the velocity exponent. *v*_max_ is the maximum velocity considered in the fit. The fit correlation factor is *R*. *G* is the interfacial thermal conductance per unit area in units of 10^–3^ W m^–2^ K^–1^.

Let us concentrate first on the homobilayers. In general, we find that as the rigidity of the system increases, λ approaches unity, and the viscous coefficient decreases. In fact, λ = 0.9 (η = 0.14) for H-H, becoming 0.83 (1.02) and 0.82 (5.29) for N-N and S-S, respectively. This behavior is related to the ability of the slider to thermalize against the substrate, a feature that depends on the rigidity. As a demonstration, one can see from panels b–d of Figure 2 that at any given velocity the slider temperature is larger for the softer layer, with H-H showing temperatures rather close to 300 K (the temperature of the substrate). The increased temperature of the slider indicates that the CM kinetic energy is efficiently converted into internal thermal energy, leading to friction.

As mentioned above, S-S displays an anomalous high temperature for *v* ≳ 550 m/s, where *f* abruptly deviates from eq 1; namely, as the slider is put in motion at a rate of 800 m/s, its internal temperature rapidly increases. This is because *f* is large, and the vibration-energy flux injected into the slider exceeds the dissipative flux to the substrate. The latter is limited by the thermal coupling between the two layers. As the friction is enhanced by temperature (see Figure S5), it causes a rapid decrease in the slider velocity, which in turn decreases the injected thermal flux. Thus, as the velocity decreases, one reaches a steady state in which the frictional energy injected is equal to the heat flow across the interface, restoring eq 1. A similar behavior is found also for N-N at *v* ≥ 1000 m/s.

The low-velocity friction can be extrapolated from eq 1 as *f* ∝ *ηλv*, where *ηλ* is effectively the Stokes friction coefficient. These are reported in Table 1, which clearly shows their severe dependence on the rigidity of the system. In fact, doubling the stiffness leads to an ∼6-fold decrease in friction. Recalling that all bilayers share an identical ISES, we conclude that the difference in friction is solely related to the internal dynamics of the layers and thermal coupling. Furthermore, by construction, all bilayers present an identical Γ-point breathing mode, which is associated with the rigid oscillation of the interlayer distance (see Figure S3). The kinetic energy associated with such a mode, extracted from the CM vertical dynamics, remains unchanged over the simulation time; namely, it is not responsible for storing energy during the motion. There is also little change in the interlayer distance, with a maximum increase of 0.015 Å found for S-S at *v* > *v*_max_. This is consistent with the observed temperature profile, and it is not caused by scattering at the ISES.

At this point, we may attempt to compare our results with experiments. This is rather difficult because most experiments measure the friction between an oscillating AFM tip and a 2D substrate and not that between two flakes. Furthermore, even when two flakes are considered, the friction force appears to be dependent on the flake size, while the mutual orientation between the flakes is usually unknown. With all this considered, experimental works report friction forces per unit area ranging between 3 and 120 kPa.^11,38,39^ We can estimate the same quantity by using the low-velocity limit of eq 1, which returns 2.9 kPa for a sliding velocity of 1 m/s. Considering that the typical sliding velocities are certainly <1 m/s, we can then conclude that our computed friction is significantly lower than that measured experimentally. A number of reasons can account for such disagreement. First, one has to note that experiments are usually performed under nonpristine conditions, where defects and contaminations play a significant role. Furthermore, due to the limitations in the exfoliation process, the nanoflakes used in experiments are not large enough to ignore shape and edge effects. Finally, the mutual orientation between the layers is largely unknown. Our simulations, instead, consider the case of infinite and incommensurate superlattices of intact 2D materials. As such, we expect that our simulations will return the lowest achievable superlubricity limit and the computed friction force to be systematically smaller than the measured one. Note also that the use of a more sophisticated description of the dispersive forces may improve the quantitative description.

Turning our attention to heterostructures combining layers of different stiffness, Figure 3 shows both *f*(*v*) and *T*(*v*) for all six possible combinations. Starting from S-N and N-S, it is clear that *f* is independent of the order of the layers for *v* < 500 m/s, while for *v* > 500 m/s, the soft slider is associated with a slightly larger friction. A more noticeable difference can be found in *T*(*v*) at large *v* values, where a soft slider moving on a normal substrate reaches 360 K, while for the opposite situation, *T* < 330 K. More generally, the soft slider is constantly hotter than the normal one when *v* > 500 m/s. Notably, in our classical MD simulations at high *T* values, all monolayers display similar heat capacities, meaning that the different *T*(*v*) profiles for S-N and N-S must be associated with a different heat flux. This develops despite the almost identical frictional forces. The variation in heat flux becomes negligible as the velocity is decreased below ∼500 m/s.

**Figure 3:**
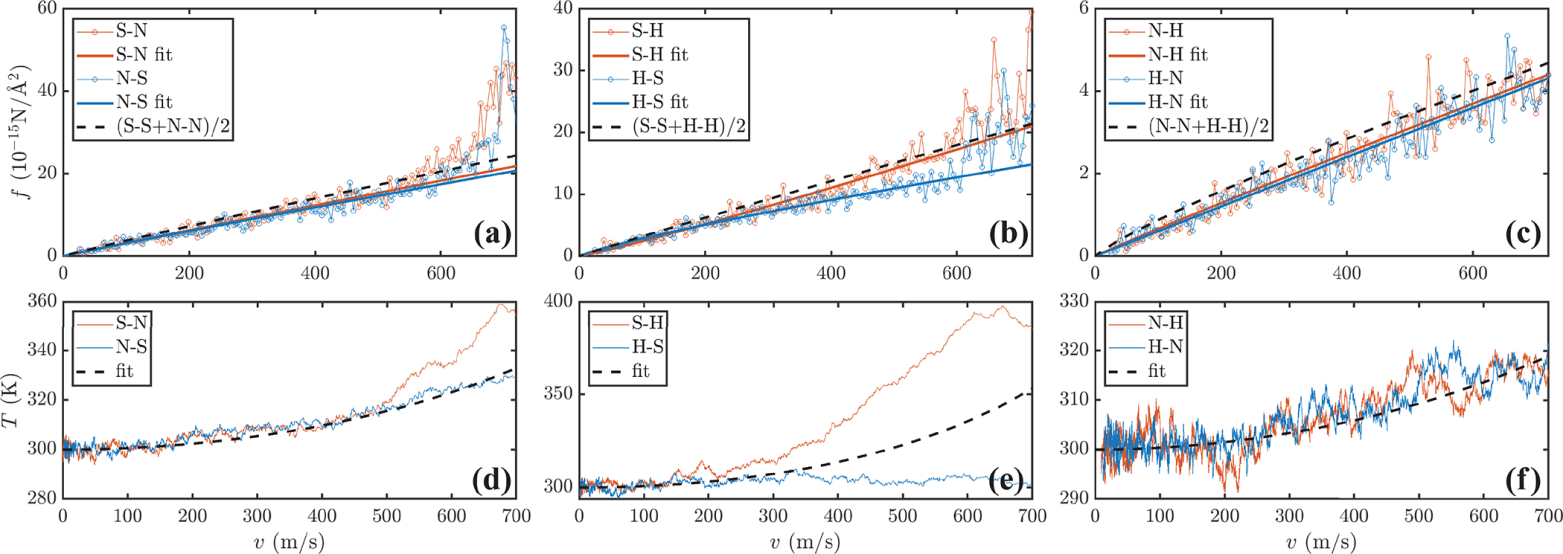
(a–c) Friction and (d–f) slider temperature as a function of the slider velocity for the six different heterobilayers investigated. The MD data (thin lines and circles) are fitted to eq 1 (solid thick lines). The dashed lines in the *f*(*v*) plots correspond to the average friction fitted to eq 1 for the corresponding homobilayers, while those in the temperature plots are calculated by using eq 2.

From Figure 3, we note that *f*(*v*) for heterobilayers is always smaller than the average friction of the associated homobilayers (dashed black lines), with the difference increasing at high *v* values. Correspondingly, the temperature gap between the slider and the substrate (kept at 300 K) in heterobilayers is significantly larger than in homobilayers. These two facts suggest that the thermal coupling between the layers in heterobilayers is weaker than in homobilayers. The friction can then be taken as the sum *f* = *f*_α_ + *f*_β_ + *f*_*αβ*_, where *f*_α_ (*f*_β_) originates from the energy dissipation to the slider (substrate) resulting from the ISES, while *f*_*αβ*_ describes phonon–phonon scattering across the layers. Because all bilayers share identical ISESs, *f*_α_ and *f*_β_ must remain unchanged with the bilayer composition. Hence, in a heterobilayer, *f* remains lower than the average of the associated homobilayers, because *f*_*αβ*_ is smaller for heterobilayers. This is a direct consequence of the reduced phonon spectral overlap between monolayers with different stiffnesses (see Figure S3).

An extreme situation is encountered for the S-H and H-S systems (Figure 3b,e). Now the high-speed temperature increase is large for S-H (≤400 K) and minimal for H-S, indicating that the main dissipation channel is through the soft layer. Because only the substrate is thermalized, such a feature results in two distinctly different *T*(*v*) profiles, depending on the order of the layers. If we assume *f*_SH_ and *f*_H_ are much smaller than *f*_S_ and compare the low-*v f*(*v*) traces of the S-H and S-S systems, we conclude that *f*_SS_ contributes to ∼30% of the total friction of S-S; namely, it is similar to *f*_S_ over the entire *v* range. Finally, N-H and H-N show similar friction and temperature profiles, resembling the S-H case at low velocities. As the layers are both relatively rigid, we do not observe any significant heating at any velocity.

Our analysis can be made quantitative by determining the interfacial thermal coupling between the layers, namely, the interfacial thermal conductance, *G*. This is extracted from the time evolution of the heat flux of layers initially thermalized at different temperatures, and its values are reported in Table 1 (see Figure S4). In general, *G* depends on the relative strengths of the inter- and intralayer interactions, namely, the binding energy and the bond stiffness. Heat is transferred across the layers when the motion of the atoms in one layer drives the motion in the other. As the interlayer van der Waals forces are identical for all heterostructures, here only the layer stiffness differentiates the thermal coupling. Thus, for homobilayers, we find *G* to be relatively large and to decrease as the layer stiffness increases. In contrast, in heterobilayers, *G* is also determined by the relative thermal impedances, in a manner similar to that described by the “acoustic mismatch model”.^40^ The two factors of relative and absolute thermal impedances combine to make the N-H bilayer the least conductive one and the S-N layer the most.

We are now in a position to complete our analysis. During the sliding process, a heat flux, *vf*, is injected into the two layers. A steady-state situation is maintained when the amount of heat transferred across the layers is constant, and it is balanced by the heat flux to the thermostat in the substrate. Then, we have
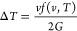
2where *f* ∝ *T*^1.6^, as extracted for the N-N bilayer by fitting eq 1 at different *T* values (see also Figure S5). The factor 2 in the denominator originates from the assumption that the heat flux splits equally between the two layers. The calculated *T*(*v*) profiles are then plotted in panels b–d of Figure 2 and panels d–f of Figure 3 as dashed lines [because the substrate is thermostated, *T*(*v*) = Δ*T*]. The curves show excellent agreement between the measured temperature and that computed from eq 2 for both homo- and heterobilayers. This means that, when *v* < *v*_max_, there is steady-state heat flux across the layers, which is then broken at higher velocities, when severe heating is observed.

In summary, *ab initio* accurate force fields have been used to investigate the superlubricant state of MoS_2_ bilayers. These have different in-plane stiffnesses but identical ISESs, a construction that allows us to investigate the sole effect of the stiffness on friction. In general, we find *f* ∝ *v*^λ^ with the exponent remaining close to unity for rigid layers and deviating for soft layers and heterogeneous bilayers. In homobilayers, a change in stiffness by a factor of 2 results in an ∼6-fold friction variation. Heterobilayers follow a similar trend, although the low-velocity friction remains small. In general, we find that the vertical breathing motion of the layers is not a major energy dissipation channel.

The thermal coupling between the layers determines the heating during the sliding process. At low velocities, a steady state is established, where the temperature difference between the slider and the substrate sustains a constant heat flux. In this regime, the slider *f* and *T* are independent of whether the slider is softer or harder than the substrate. In contrast, at higher temperatures, the slider can be heated, with the effect being much more pronounced for soft sliders. The crossover between these two regimes is dependent on the specific layer combinations. In particular, we find that heterobilayers composed of rigid materials can sustain ultralow friction and moderate heating even at extremely high velocities.

## Supporting Information Available

Computational details of SNAP generation, phonon band structures of MoS_2_ bilayers studied in this work, supercell configuration of the 21.8° twisted lattices, interfacial thermal conductance of the bilayers, and temperature dependencc of viscous cofficient η (PDF)
Transparent Peer Review report available (PDF)
