# Supplementary material for: Stiffness and Atomic-Scale Friction in Superlubricant MoS2 Bilayers
Source: J Phys Chem Lett. 2023 Jun 26;14(26):6086–91. doi: 10.1021/acs.jpclett.3c01066 (PMC10331825; doi:10.1021/acs.jpclett.3c01066)
Supplement: Supplementary file 1 — jz3c01066_si_001.pdf [file jz3c01066_si_001.pdf]

Supporting Information: Stiffness and Atomic-Scale Friction in Superlubricant MoS_2 Bilayers

Rui Dong,* Alessandro Lunghi, and Stefano Sanvito

School of Physics, AMBER and CRANN Institute, Trinity College, Dublin 2, Ireland

E-mail: dongru@tcd.ie

Generation of the SNAP for MoS_2

A spectral neighbor analysis potential (SNAP) is created for the MoS_2 monolayer using density functional theory (DFT) simulations. The training set contains 720 configurations of a 3×3 supercell of MoS_2 monolayers. Among them, 400 configurations correspond to the original lattice without strain. The x, y and z coordinates of all atoms are then randomly displaced in 4 ways:

1. A maximum displacement of δ=0.025Å is applied to all atoms,
2. A maximum displacement of δ=0.05Å is applied to all atoms,
3. A maximum displacement of δ=0.075Å is applied to all atoms,
4. A maximum displacement of δ=0.1Å is applied to two randomly selected atoms.

The remaining 320 configurations correspond to the lattice under strain. In particular, we consider 16 nonequivalent deformations, with strain tensor ε_{ij} applied as follows:

1. ε_{11}=ε_{22}=-5%, -3%, 3%, 5%,

2. ε_{11}=-5%, -3%, 3% and 5% and ε_{22}=0,

3. ε_{22}=-5%, -3%, 3% and 5% and ε_{11}=0,

4. ε_{12}=ε_{21}=-0.03, -0.015, 0.015 and 0.03.

The configurations are then obtained by random displacement of the atoms with a maximum amplitude of δ=0.05 of the strained lattices. DFT simulations are carried out with VASP[2] using the PBE exchange-correlation functional and projector augmented wave (PAW) pseudo-potentials. The energy cutoff is 500 eV and the k-point mesh is 18×18×1 for unit-cell calculations. The k-point mash converts to 6×6×1 for the 3×3 supercell.

The potential is then fitted by using the atomic coordinates and the total energy of the 720 configurations. In order to verify the accuracy of the SNAP potential, a molecular dynamics (MD) simulation is carried out for a 3×3 supercell at a temperature of 300 K. Snapshots of the atoms are taken every 10 ps for 1 ns, after an equilibration of 0.5 ns. The resulting 100 configurations form the test set. The SNAP-predicted total energies and atomic forces of the test sets are compared against the DFT values, and the results are shown in Fig. S1. Although the forces are not used for the fitting, we compare the forces calculated by SNAP with the DFT ones on the training set. The root-mean-square errors of energies and forces are listed in Table 1.

Table 1: The root-mean-square error (RMSE) of total energies and forces over the training and test sets. ΔE is the RMSE of energy and the unit is meV/atom. Δf is the RMSE of the x, y and z components of atomic forces and the unit is meV/Å. The SNAP is only fitted with total energies.

	ΔE (meV/atom)	Δf (meV/Å)
Training	0.64	0.37
Test	1.04	0.21

In Fig. S2 we compare the phonon band-structure of monolayer MoS_2 computed with

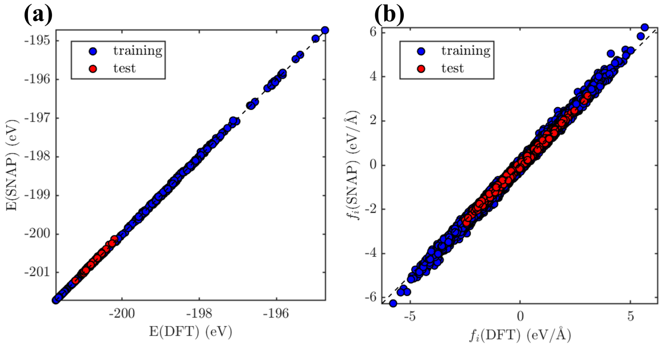

Figure S1: Parity plot of the SNAP predicted energies and forces against the DFT ones (GGA-PBE). Training set data are in blue, test set in red.

both SNAP and DFT at the GGA-PBE level (the same electronic structure theory used to generate the SNAP training set). The mean average deviation of the SNAP frequencies from the DFT ones over the high-symmetry directions is 0.25 THz.

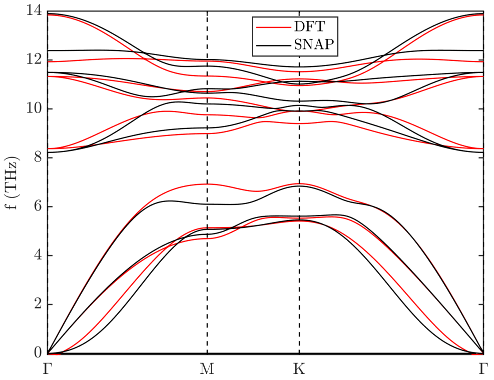

Figure S2: Phonon spectra for MoS_2 monolayer computed from finite difference with both SNAP and DFT at the GGA-PBE level.

To further validate the potential, we calculate the elastic constant of monolayer MoS_2 and compare with DFT result. For a hexagonal 2D lattice, c_{11} and c_{12} are the only two independent elements in the elastic tensor. The results are listed in Table 2.

The inter-layer interaction is computed through a Lennard-Jones (LJ) potential. The C_6 coefficients are extracted from the Tkatchenko-Scheffler van der Waals (TS-vdW) corrections with iterative Hirshfeld partitioning. FHI-AIMS[3] is used with the "tight" version of its

Table 2: Elements of elastic tensor of monolayer MoS_2. Unit is eV/Å^2

	c_{11}	c_{12}
DFT	224.2	50.2
SNAP	245.4	52.9

numerical atom-centered orbits. The extracted LJ coefficients are listed in Table 3. To test the interlayer potential we calculate the inter-layer sliding energy surface (ISES) of bilayer MoS_2 using both DFT and the LJ potential, which return us an ISES corrugation of 23.3 meV/Å^2 and 16.3 meV/Å^2, respectively.

Table 3: LJ coefficient of Mo and S in monolayer MoS_2.

	ϵ	r_0
Mo-Mo	1.0624	3.878597
Mo-S	0.4124	3.75114
S-S	0.198443	3.62368

Phonon bandstructure

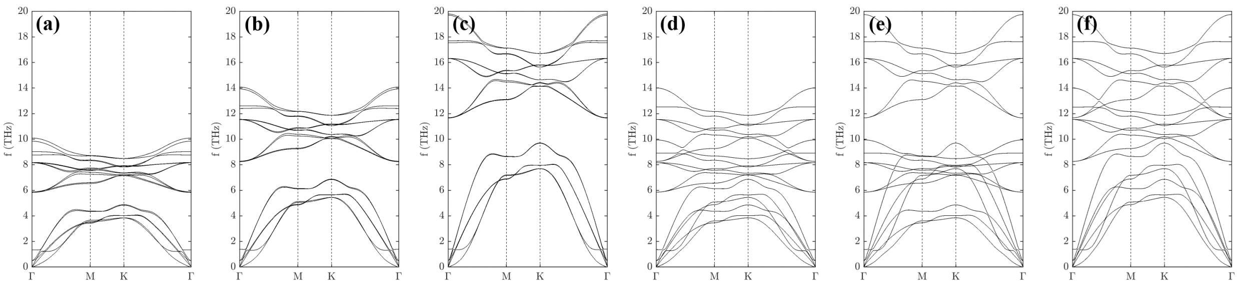

Figure S3: Phonon spectra of combinations of soft (S), normal (N) and hard (H) MoS_2 layers. (a) S-S, (b) N-N, (c) H-H, (d) S-N, (e) S-H and (f) N-H.

The phonon spectra of bilayers are calculated with LAMMPS[?] using the finite different method. A 6×6×1 super-cell is used for the MoS_2 bilayer. Each atom in the primitive cell is displaced along the x-, y- and z-axis by 0.01 Å, and the energies are calculated by using our constructed spectral neighbor analysis potential (SNAP) and the inter-layer Lennard-Jones potential, as described in the main text. Fig. S3(a)-(c) present the intra-layer stiffness of soft (S), normal (N) and hard (H) MoS_2 bilayers. Fig. S3(d)-(f) show the different level of overlap between the phonon band-structures of hetero-bilayeres. The two lowest modes at Γ are the shear mode (SM) and layer-breathing mode (LBM). One should notice they have the same frequencies for all the six combinations. This provides evidence that the ISES are the same.

Supercell configuration with twist angle 21.8°

In order to find the possible supercell configurations of two identical hexagonal 2D lattices, as shown in yellow and purple in Fig. S4, the twist angle θ needs to be searched between 0° and 30°. If we constraint the supercell to be hexagonal and without in-plane strain, the smallest supercell areas will be found for 21.8° ($\sqrt{7} \times \sqrt{7}$).

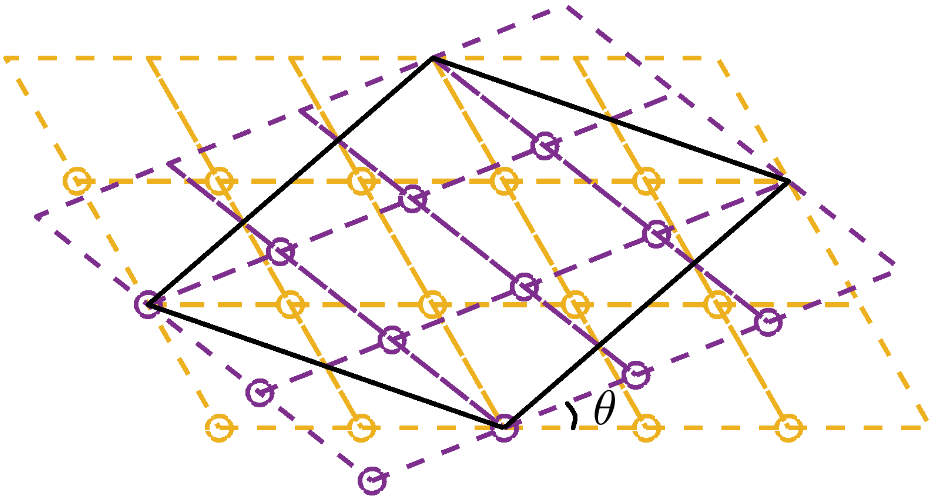

Figure S4: Sketch of supercell with twist angle 21.8°.

Calculation of the thermal conductance across the layers

Since the out-of-plane thermal conductivity of a bilayer is ill-defined, we instead compute the interfacial thermal conductance, G. This simply relates the heat flux, q, with the temperature difference ΔT, $q = G\Delta T$, and it can be extracted from MD simulations. We first equilibrate the two layers at 400 K and 200 K, respectively. In the absence of external thermal reservoirs, the temperature difference between the two layers decays exponentially in time from its initial value, ΔT_0=200 K (see Fig. S5), as

$$\Delta T(t) = \Delta T_0 \exp\left(-\frac{2G}{C_v}t\right) \,, \tag{1}$$

where C_v is the specific heat. G can then be extracted by monitoring the time-evolution of ΔT. In performing the fit C_v=75.75 J/mol·K^{-1} is calculated from the total energy fluctuations. As expected G is found not to depend on the direction of the heat flux and the computed values (per unit area) are reported in Table I in the main text.

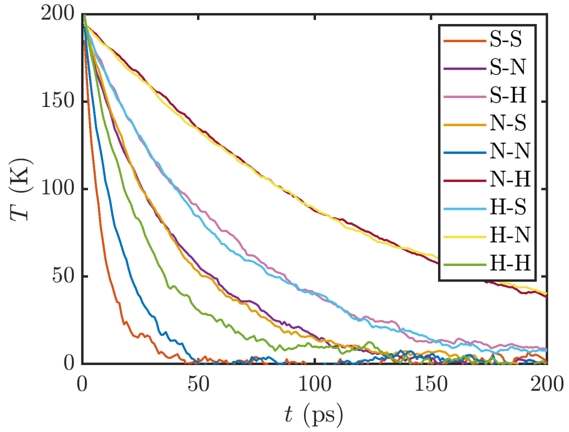

Figure S5: (Color online) Time evolution of the temperature gap between the two layers of a bilayer, following equilibration at 400 K and 200 K, respectively. Each curve is the average over 10 different trajectories.

Temperature dependence of η

In order to study how the friction depends on temperature, we simulate the free sliding of the N-N bilayer, where the substrate is thermalized at different temperatures. The substrate temperatures are set to range from 150 K to 450 K in 50 K steps. The friction force is fitted to $f = -\eta(v/v_{\text{ref}})^{\lambda}$ [see Eq. (1) in the main text]. Here k is set the value at 300 K, namely 0.83, so that η is the only parameter left to be determined. This approach eliminates numerical instabilities in the fit and leads to a better comparison between different temperatures. The $\eta(T)$ curve is then fitted again to a power-law $\eta = C \cdot T^n$, and we obtain n=1.6. Our results are shown in Fig. S4.

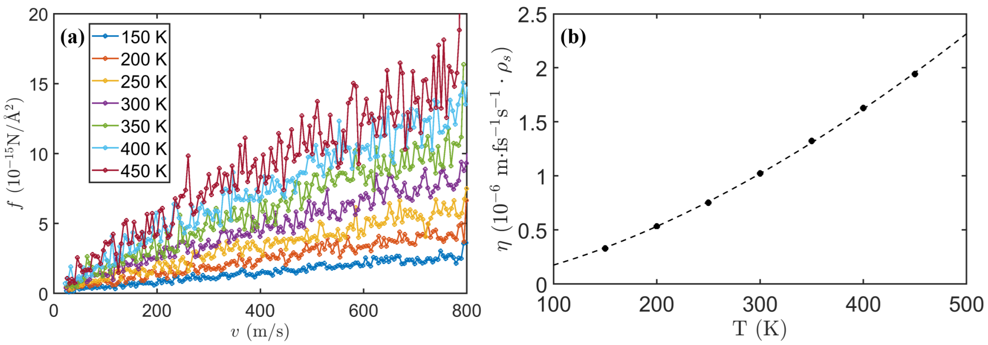

Figure S6: Temperature dependence of the friction force. (a) $f(v)$ curves for the N-N bilayer at different temperatures. (b) Fit of the viscous coefficient η to the power-law model. The solid dots are the values of η and the dashed line is the fit, $\eta(T)$.

References

(1) Thompson, A.P.; Aktulga, H.M.; Berger, R.; Bolintineanu, D.S.; Brown, W.M.; Crozier, P.S.; in't Veld, P.J.; Kohlmeyer, A.; Moore, S.G.; Nguyen, T.D.; Shan, R.; Stevens, M.J.; Tranchida, J.; Trott, C.; Plimpton, S.J. LAMMPS - a flexible simulation tool for particle-based materials modeling at the atomic, meso, and continuum scales. *Comp. Phys. Comm.*, **2022**, 271, 10817, 1-34. DOI: 10.1016/j.cpc.2021.108171

(2) Kresse, G.; Furthmüller, J. Efficiency of ab-initio total energy calculations for metals and semiconductors using a plane-wave basis set. *Comput. Mater. Sci.*, **1996**, 6(1), 15-20. DOI: 10.1016/0927-0256(96)00008-0

(3) Havu, V.; Blum, V.; Havu, P.; Scheffler M. Effcient O(N) integration for all-electron electronic structure calculation using numeric basis functions. *J. Comput. Physics*, **2009**, 228(22), 8367-8379. DOI: 10.1016/j.jcp.2009.08.008
