# Supplementary material for: Stiffness and Atomic-Scale Friction in Superlubricant MoS2 Bilayers
Source: J Phys Chem Lett. 2023 Jun 26;14(26):6086–91. doi: 10.1021/acs.jpclett.3c01066 (PMC10331825; doi:10.1021/acs.jpclett.3c01066)
Supplement: Supplementary file 2 — jz3c01066_si_004.pdf [file jz3c01066_si_004.pdf]

jz-2023-010666.R1

Name: Peer Review Information for "Stiffness and Atomic-Scale Friction in Superlubricant MoS_2 Bilayers"

First Round of Reviewer Comments

Reviewer: 1

Comments to the Author

In this work, molecular dynamics simulations performed with chemically accurate ab-initio machine-learning force fields were used to study the superlubricant

state of MoS2 bi-layers. The authors demonstrated that the layer stiffness has profound effects on the superlubricant state of two-dimensional van der Waals heterostructures, and they found that a twofold increase in the intra-layer stiffness reduces the friction by approximately a factor six. Two sliding regimes as a function of the sliding velocity were also found.

In my opinion, the topic is timely and interesting, and the manuscript is well-written. In this work, results are sound, and strongly support the conclusion. I am very happy to recommend its publication in The Journal of Physical Chemistry Letters as is.

Reviewer: 2

Comments to the Author

1. What is the major advance reported in the paper?

This manuscript presents MD simulations on the phenomenon of superlubricity in MoS2 bilayers. The main novelty consists in studying how intralayer stiffness interplays with interlayer van der Waals (vdW) interactions. The findings go beyond the traditional frozen-layer approach used in previous DFT calculations. The paper employs an interesting strategy where intralayer interactions are treated with a machine learning force field, whereas interlayer interactions are included using a Lennard-Jones potential. Overall, I find the level of advance and technical sophistication to be appropriate for publication in JPCL. I think some of the ideas could be transported beyond 2D materials. In general, the study of how covalent and non-covalent interactions interplay in complex materials is of broad interest and high impact.

2. What is the immediate significance of this advance?

That dynamical effects are important when trying to understand sliding dynamics of 2D materials and how the interplay between covalent and non-covalent interactions contributes to the friction in such interfaces.

3. Technical suggestions

I have two main suggestions:

(1) The phenomenon of superlubricity has been widely studied experimentally. However, the comparison to experiment in this study is very weak. I would like to see better qualitative and quantitative comparison. If this comparison is not good, reasons must be given of why the employed potential is insufficient. I think this lack of comparison is currently the weakest point in the manuscript.

(2) The authors employ a rather simple potential in their study, especially for the interlayer interactions. It has been demonstrated rather extensively that interlayer interactions in 2D materials are rather subtle. For example, many-body dispersion effects must be included for an accurate treatment, see https://doi.org/10.1103/PhysRevLett.114.096101 and https://doi.org/10.1103/PhysRevLett.128.106101 for graphene/hBN/nanotubes and https://doi.org/10.1021/acs.jctc.1c00782 for MoS2. I think a discussion of this fact should be provided in the manuscript and a way to address these effects described. Indeed, many-body vdW effects become more prominent for non-equilibrium geometries (https://doi.org/10.1103/PhysRevResearch.5.L012028), hence I expect that dynamical simulations should eventually include them. I wonder whether the authors share this opinion given the published evidence.

Reviewer: 3

Comments to the Author

The manuscript presented by S. Sanvito et al. approaches the superlubricant state of MoS2 bi-layers by using a machine learning methodology combined with molecular dynamics.

The manuscript is clear and well written and the topic is of broad interest. However, I would like to point some minor details to improve the text before publication:

1) In page 3, the interlayer interaction (binding energy) is described by a means of a Lennard-Jones potential. The authors point that scaling such binding energy can be related to the obtention of different stiffness structures. I would like to see a more detailed explanation on this in the main document or at least some references if such approach has been used in any previous work.

2) In page 4, the authors claim that the smallest superlubricant state is found for a geometrical twist of 21.8º. Can the authors illustrate this? Is there any atomic-interaction reason for such behavior?

3) In page 4, the starting velocity for the slider layers is set to 800m/s which is enormously high. Is there any reason for that particular value? How dramatically would the results change with a quite different starting value?

4) In page 4, Nosé-Hoover approach would need a citation.

5) In page 4,5 and 6. S, N and H notation is very unclear. Actually, it recalled the atomic nomenclature of chemical bonding and confuses the reader. I would suggest the authors to modify it to something not so chemically obvious.

6) In page 10, the concept of 'acoustic mismatch' coupled to interfacial phonons is not clear. The authors should extend the text in this point and explain in more detail what do they refer to.

Reviewer: 4

Comments to the Author

The manuscript entitled "Stiffness and atomic-scale friction in superlubricant MoS2 bilayers" used molecular dynamics simulations combing with chemically accurate ab-initio machine-learning force fields to investigate the superlubricant state of MoS2 bi-layers with different stiffnesses. They found that stiffness has a profound effect on friction, and at low velocity, the friction is independent of layer order. The thermal coupling between the layers determines heating during sliding, with hetero-bilayers composed of rigid materials sustaining ultra-low friction even at high velocities.

Overall, the manuscript is in good written and the content is clear. However, there are two significant and fundamental issues that need to be addressed, please see the followings. Addressing these issues will significantly improve the manuscript's quality and ensure that the results are credible and valuable to the scientific community. Thus, in the current stage, I cannot recommend that the manuscript be published in the Journal of Physical Chemistry Letters.

Comments

- In the molecular dynamics investigation section, the authors utilized the LAMMPS software for calculations. However, it is unclear how they implemented ab-initio machine-learning force fields calculations. Based on the computational details, it appears that the authors may have used the Vienna Ab-initio Simulation Package (VASP). If this is the case, it's worth noting that the latest version of VASP (version 6.4.1) includes many enhancements and bug fixes related to machine-learning force fields, which could potentially address issues that may have existed in previous versions such as memory-related problems. It is essential for the authors to address how they verified the accuracy of their calculations, given the potential issues that may have arisen from using previous versions of the software. Therefore, it is important for the authors to provide information on their validation and verification procedures to ensure that their results are reliable and reproducible.

- Another technical issue that needs to be addressed is the type of van der Waals (vdW) correction used in the calculations. In the field of ab initio calculations, there are different types of vdW corrections that can be used, such as nonlocal DF-type vdW correction (originally proposed by Dio et al. published in PRL 92 (2004) 246001), which includes DF, DF2, DF3, optPBE, rVV10, and others, as well as semi-empirical D-type vdW correction, which includes D2, D3, D4, Tkatchenko-Scheffler method, and others. The details of the vdW correction used in the calculations are not provided in the manuscript, which makes it difficult to assess the validity of their results. Therefore, it is important for the authors to provide information on the vdW correction they used and how they validated their results to ensure that their calculations are accurate and reliable.

Author's Response to Peer Review Comments:

Dear Prof. Editor,

We thank your office for handling the review of our manuscript, and the reviewers for their reports, which give us the opportunity to improve our manuscript. We are pleased to note that all four referees evaluate positively our work.

Below we address each reviewers' comments point-by-point and indicate the modifications made to the revised manuscript. For convenience, the referees' comments are in black, our reply in blue and the changes made to the manuscript in red. As you will see, we have responded to all referees' suggestions and we believe that the manuscript is now suitable for publication in the Journal of Physical Chemistry Letters.

Thank you for your consideration, Best regards,

Rui Dong, Alessandro Lunghi and Stefano Sanvito

Reviewer: 1

Recommendation: This paper represents a significant new contribution and should be published as is.

Comments: In this work, molecular dynamics simulations performed with chemically accurate ab-initio machine-learning force fields were used to study the superlubricant state of MoS2 bi-layers. The authors demonstrated that the layer stiffness has profound effects on the superlubricant state of two-dimensional van der Waals heterostructures, and they found that a twofold increase in the intra-layer stiffness reduces the friction by approximately a factor six. Two sliding regimes as a function of the sliding velocity were also found.

In my opinion, the topic is timely and interesting, and the manuscript is well-written. In this work, results are sound, and strongly support the conclusion. I am very happy to recommend its publication in The Journal of Physical Chemistry Letters as is.

Reply: We thank the reviewer for reading our manuscript and for their appreciation of our work.

Additional Questions:
Urgency: Top 10%
Significance: Top 10%
Novelty: Top 10%
Scholarly Presentation: Top 10%
Is the paper likely to interest a substantial number of physical chemists, not just specialists working in the authors' area of research?: Yes

Reviewer: 2

Recommendation: This paper may be publishable, but major revision is needed; I would like to be invited to review any future revision.

Comments: 1. What is the major advance reported in the paper?
This manuscript presents MD simulations on the phenomenon of superlubricity in MoS2 bilayers. The main novelty consists in studying how intralayer stiffness interplays with interlayer van der Waals (vdW) interactions. The findings go beyond the traditional frozen-layer approach used in previous DFT calculations. The paper employs an interesting strategy where intralayer interactions are treated with a machine learning force field, whereas interlayer interactions are included using a Lennard-Jones potential. Overall, I find the level of advance and technical sophistication to be appropriate for publication in JPCL. I think some of the ideas could be transported beyond 2D materials. In general, the study of how covalent and non-covalent interactions interplay in complex materials is of broad interest and high impact.

2. What is the immediate significance of this advance?
That dynamical effects are important when trying to understand sliding dynamics of 2D materials and how the interplay between covalent and non-covalent interactions contributes to the friction in such interfaces.

Reply: We first want to thank the reviewer for their critical reading of our manuscripts and for the suggestions, which have helped in improving our work. Here we reply in detail to the two criticisms made.

3. Technical suggestions. I have two main suggestions:
(1) The phenomenon of superlubricity has been widely studied experimentally. However, the comparison to experiment in this study is very weak. I would like to see better qualitative and quantitative comparison. If this comparison is not good, reasons must be given of why the employed potential is insufficient. I think this lack of comparison is currently the weakest point in the manuscript.

Reply: In the original manuscript, we did not include a comparison with existing experimental literature, mostly because we were interested in providing a proof of principle, but also because our work analyzes a velocity range at the extreme limit of what can be achieved experimentally. Nevertheless, the question is interesting and, we agree, deserves attention.
In general a comparison with experiments is not simple, since the friction coefficient is typically computed between an AFM tip and a 2D material and not between two monolayers. Furthermore, in experiments the frictional force is measured as a function of the load (usually estimated), namely differently from what proposed here. All this considered, we believe that the most relevant experimental work to compare with is that contained in reference [Li, H.; Wang, J.; Gao, S.; Chen, Q.; Peng, L.; Liu, K.; Wei, X. Superlubricity between MoS2 Monolayers. *Adv. Mat.*, **2017**, 29(27), 1701474, 1-6.], where the lateral sliding forces between two MoS_2 flakes are measured. These depend on the flake size and the authors report a range of forces per unit area (shear strengths) comprised between 23kPa and 120kPa. We can estimate the same quantity by using the "$\eta\lambda$" coefficient (our extrapolation to low velocity) and assume a velocity of 1 m/s (unfortunately the sliding velocity used in the experiments is not known). This estimate returns us a shear strength of 2.9kPa, which is significantly smaller than what measured in experiments. Subsequent experimental data [see Liao, M. *et al.*, *Ultra-low friction and edge-pinning effect in large-lattice-mismatch van der Waals heterostructures. Nat. Mater., 2022, 21, 47-54.*] suggest that shear strengths as low as 3kPa can be measured in MoS_2, but these values are obtained for a velocity significantly lower than 1m/s. As such, we should conclude that our estimated friction is typically smaller than what measured.

In addition to the differences arising from the different ways of estimating the friction, we believe that there is a number of reasons for such discrepancy. Firstly, it is important to note that experiments are usually performed in non-pristine conditions, where defects (mostly S vacancies in mechanically exfoliated flakes) and contaminations play a significant role. Furthermore, due to limitations in fabrication technology, the nano-flakes commonly used in experiments are not large enough to ignore the effect of shape and edges. Our simulations, instead, consider the case of infinite and commensurate super-lattices of intact 2D materials. This allows us to identify the intrinsic limit of friction in these compounds and provides a guide to what limit can be expected for this property. We have added a discussion about the comparison to experiments in the main text. The new paragraph reads:

At this point we may attempt to compare our results with experiments. This is rather difficult, since most experiments measure the friction between an oscillating AFM tip and a 2D substrate and not between two flakes. Furthermore, even when two flakes are considered, the friction force appears to be dependent on the flake size while the mutual orientation between the flakes is usually unknown. With all this considered experimental works report friction forces per unit area ranging between 3 kPa and 120 kPa [11,38,39]. We can make an estimate for the same quantity by using the low-velocity limit of Eq. (1), which returns us 2.9 kPa for a sliding velocity of 1 m/s. Considering that the typical sliding velocities are certainly slower than 1 m/s, we can then conclude that our computed friction is significantly lower than what measured experimentally. A number of reasons can account for such disagreement. Firstly, one has to note that experiments are usually performed in non-pristine conditions, where defects and contaminations play a significant role. Furthermore, due to the limitations in the exfoliation process, the nano-flakes used in experiments are not large enough to ignore shape and edge effects. Finally, the mutual orientation between the layers is largely unknown. Our simulations, instead, consider the case of infinite and incommensurate super-lattices of intact 2D materials. As such, we expect that our simulations will return the lowest achievable superlubricity limit, and the computed friction force to be systematically smaller than the measured one.

with

(38) Wang, J.; Cao, W.; Song, Y.; Qu, C.; Zheng, Q.; Ma, M. Generalized Scaling Law of Structural Superlubricity. *Nano Lett.*, **2019**, 19(11), 7735-7741.

(39) Liao, M.; Nicolini, P.; Du, L.; Yuan, J.; Wang, S.; Yu, H.; Tang, J.; Cheng, P.; Watanabe, K.; Taniguchi, T.; Gu, L.; Claerbout, V.E.P.; Silva, A.; Kramer, D.; Polcar, T.; Yang, R.; Shi, D.; Zhang, G. Ultra-low friction and edge-pinning effect in large-lattice-mismatch van der Waals heterostructures. *Nat. Mater.*, **2022**, 21, 47-54.

(2) The authors employ a rather simple potential in their study, especially for the interlayer interactions. It has been demonstrated rather extensively that interlayer interactions in 2D materials are rather subtle. For example, many-body dispersion effects must be included for an accurate treatment, see https://doi.org/10.1103/PhysRevLett.114.096101 and https://doi.org/10.1103/PhysRevLett.128.106101 for graphene/hBN/nanotubes and https://doi.org/10.1021/acs.jctc.1c00782 for MoS2. I think a discussion of this fact should be provided in the manuscript and a way to address these effects described. Indeed, many-body vdW effects become more prominent for non-equilibrium geometries (https://doi.org/10.1103/PhysRevResearch.5.L012028), hence I expect that dynamical simulations should eventually include them. I wonder whether the authors share this opinion given the published evidence.

Many thanks to the reviewer for pointing this out. It is certainly true that simple LJ-type potentials sometimes fail to represent the inter-layer interaction accurately. This issue seems to be more

noticeable for polar 2D materials, such as h-BN, and for graphene. For example, there are examples of large discrepancy between DFT-computed inter-layer sliding energy surfaces (ISES) and those obtained with inter-layer potentials (as from the references suggested by the referee).

In our case, we have compared the corrugation of the ISES of MoS_2 computed with either the LJ potential or DFT using the Tkatchenko-Scheffler van der Waals (TS-vdW) corrections with iterative Hirshfeld partitioning. For the first we find a value of 16.3 meV/A^2, while the second returns us 23.3 meV/A^2. This suggests that the chosen LJ potential produces an ISES of quality close to the more complex DFT calculations. We believe that this is sufficient to show the effects discussed in our manuscript (the role of the materials stiffness on superlubricity), in particular since we do not investigate the dependence of the friction on the number of layers, for which a good handle of screening is necessary.

We have added a note in the Supporting Information (SI) on the results mentioned above and the correct pointer into the main manuscript. The note reads:

The inter-layer interaction is computed through a Lennard-Jones (LJ) potential. The C6 coefficients are extracted from the Tkatchenko-Scheffler van der Waals (TS-vdW) corrections with iterative Hirshfeld partitioning. FHI-AIMS is used with the "tight" version of its numerical atom-centered orbits. The extracted LJ coefficients are listed in Table III. To test the interlayer potential we calculate the inter-layer sliding energy surface (ISES) of bilayer MoS2 using both DFT and the LJ potential, which return us an ISES corrugation of 23.3 meV/˚A^2 and 16.3 meV/˚A^2, respectively.

Additional Questions:
Urgency: High
Significance: High
Novelty: High
Scholarly Presentation: High
Is the paper likely to interest a substantial number of physical chemists, not just specialists working in the authors' area of research?: Yes

Reviewer: 3

Recommendation: This paper is publishable subject to minor revisions noted. Further review is not needed.

Comments: The manuscript presented by S. Sanvito et al. approaches the superlubricant state of MoS2 bi-layers by using a machine learning methodology combined with molecular dynamics. The manuscript is clear and well written and the topic is of broad interest. However, I would like to point some minor details to improve the text before publication:

We thank the reviewer for reading our manuscript and offering their suggestions. Our replies are as follows.

1) In page 3, the interlayer interaction (binding energy) is described by a means of a Lennard-Jones potential. The authors point that scaling such binding energy can be related to the obtention of different stiffness structures. I would like to see a more detailed explanation on this in the main document or at least some references if such approach has been used in any previous work.

We apologize for the confusion that we may have created. The scaling factors 2 and 1/2 are applied only to compute the intra-layer potential, namely the SNAP, but are not applied to the LJ one. The advantage of scaling SNAP is that we can tune the stiffness of the material without altering the binding energy and the geometry minimum. To avoid confusion we have now moved the description of the inter-layer LJ potential to the end of the paragraph.

2) In page 4, the authors claim that the smallest superlubricant state is found for a geometrical twist of 21.8º. Can the authors illustrate this? Is there any atomic-interaction reason for such behavior?

We have added a demonstration of the 21.8° superlattice angle in the Supporting Information (SI). The magical angle of 21.8° comes from pure geometrical reasons. To find supercell configurations of two identical hexagonal 2D lattices, the twist angle needs to be searched between 0° and 30°. The smallest supercell areas are found for 21.8° ($\sqrt{7} \times \sqrt{7}$), 27.8° ($\sqrt{13} \times \sqrt{13}$), 13.2° ($\sqrt{19} \times \sqrt{19}$) and 10.9° ($3\sqrt{3} \times 3\sqrt{3}$). The new paragraph describing the 21.8° superlattice angle reads (note that a figure has also been included in the new SI):

In order to find the possible supercell configurations of two identical hexagonal 2D lattices, as shown in yellow and purple in Fig. S4, the twist angle θ needs to be searched between 0° and 30°. If we constraint the supercell to be hexagonal and without in-plane strain, the smallest supercell areas will be found for 21.8° ($\sqrt{7} \times \sqrt{7}$).

3) In page 4, the starting velocity for the slider layers is set to 800m/s which is enormously high. Is there any reason for that particular value? How dramatically would the results change with a quite different starting value?

Due to the ultra flat inter-layer sliding energy surface (ISES), the layers perform Brownian motion at ~30 m/s in equilibrium at room temperature. This prevents us from reaching the typical sliding velocities considered in tribology experiments (note that in experiments the layer motion is constrained by the AFM tip). The velocity of the Brownian motion approaches zero when the size of layer/flake is large enough. However, such large system is unreachable by the current simulation method.

In our case, the simulation requirements are to maximize the velocity range so to obtain as many data points as possible, and to maintain a "steady" state. If the starting velocity is too high, this will trigger the "heat-up" phenomenon mentioned on page 11 of the manuscript. The value of 800 m/s is chosen for normal MoS_2 bilayer according to these rules. Note that we also did test the use of different

starting velocities in the normal MoS_2 bilayer case, for example, 600 m/s and 400 m/s. The force vs. velocity curves obtained coincide with the one reported in the paper.

4) In page 4, Nosé-Hoover approach would need a citation.

The citation has now been added.

(37) Shinoda, W.; Shiga, M.; Mikami, M. Rapid estimation of elastic constants by molecular dynamics simulation under constant stress. Phys. Rev. B, 2004, 69(13), 134103, 1-8.

5) In page 4,5 and 6. S, N and H notation is very unclear. Actually, it recalled the atomic nomenclature of chemical bonding and confuses the reader. I would suggest the authors to modify it to something not so chemically obvious.

We appreciate that there may be some confusion. However, using longer definitions (e.g. "norm" for "normal") will make the legends of the figure quite long, losing space for the data themselves. As such, we prefer to remain with our original definitions.

6) In page 10, the concept of 'acoustic mismatch' coupled to interfacial phonons is not clear. The authors should extend the text in this point and explain in more detail what do they refer to.

The acoustic mismatch model is used to calculate the interfacial acoustic reflectance

$$r(\omega) = \frac{Z_A(\omega) - Z_B(\omega)}{Z_A(\omega) + Z_B(\omega)}$$

where $Z(\omega)$ is the acoustic impedance of material A and B. This model also works for thermal impedance and interfacial conductance. The out-of-plane thermal conductivity is ill-defined in 2D materials, therefore, the formula does not directly apply to our hetero bilayer MoS_2. The sentence at page 10 suggests that there is a factor $\frac{k_A - k_B}{k_A + k_B}$ in determining the conductance G, where k_A and k_B are the stiffness of the monolayers.

We have now modified the text to make it clearer. It now reads:

In contrast, in hetero-bilayers, G is also determined by the relative thermal impedances, in a way similar to that described by the "acoustic mismatch model".[40] The two factors of relative and absolute thermal impedances combine to make the N-H bi-layer to be the least conductive one and the S-N to be the most.

with

Wei, Fan Jun and Mole, Richard A. and Karna, Sunil K. and Shi, Jin Wei and Sheu, Jinn Kong and Lin, Kung Hsuan, Applied Physics Letters **15**, 114 (2019)

Additional Questions:
Urgency: Moderate
Significance: High
Novelty: High
Scholarly Presentation: Top 10%
Is the paper likely to interest a substantial number of physical chemists, not just specialists working in the authors' area of research?: Yes

Reviewer: 4

Recommendation: This paper may be publishable, but major revision is needed; I would like to be invited to review any future revision.

Comments: The manuscript entitled "Stiffness and atomic-scale friction in superlubricant MoS2 bilayers" used molecular dynamics simulations combing with chemically accurate ab-initio machine-learning force fields to investigate the superlubricant state of MoS2 bi-layers with different stiffnesses. They found that stiffness has a profound effect on friction, and at low velocity, the friction is independent of layer order. The thermal coupling between the layers determines heating during sliding, with hetero-bilayers composed of rigid materials sustaining ultra-low friction even at high velocities.

Overall, the manuscript is in good written and the content is clear. However, there are two significant and fundamental issues that need to be addressed, please see the followings. Addressing these issues will significantly improve the manuscript's quality and ensure that the results are credible and valuable to the scientific community. Thus, in the current stage, I cannot recommend that the manuscript be published in the Journal of Physical Chemistry Letters.

We thank the reviewer for reading our manuscript, and we apologize for missing a few important details in the generation of the potential. The replies to each comment are presented here.

Comments: In the molecular dynamics investigation section, the authors utilized the LAMMPS software for calculations. However, it is unclear how they implemented ab-initio machine-learning force fields calculations. Based on the computational details, it appears that the authors may have used the Vienna Ab-initio Simulation Package (VASP). If this is the case, it's worth noting that the latest version of VASP (version 6.4.1) includes many enhancements and bug fixes related to machine-learning force fields, which could potentially address issues that may have existed in previous versions such as memory-related problems. It is essential for the authors to address how they verified the accuracy of their calculations, given the potential issues that may have arisen from using previous versions of the software. Therefore, it is important for the authors to provide information on their validation and verification procedures to ensure that their results are reliable and reproducible.

We apologize for the confusion in describing the potential generation. Indeed, we used VASP in the calculation of training and test sets, but we didn't use the "on-the-fly" machine-learning algorithm included in VASP to generate the force field.

Our own scripts generate the geometries of the training set. These are then converted to the POSCAR format and passed to VASP to calculate the total energies and atomic forces. The energies and forces and their corresponding geometries are then used to fit the SNAP, again by using our own codes. The generated SNAP is used to calculate the energies and forces of the configurations in the training set. Results are compared to DFT through the evaluation of the root-mean-square errors (RMSEs) in training set.

Then, we run MD simulations with LAMMPS, with the newly generated SNAP. Snapshots are extracted from the MD trajectories, converted into POSCAR format and passed to VASP to evaluate total energies and forces. These data form the test set. Finally, we compare the DFT and SNAP results to calculate the RMSE for the test set. Both training and test errors are now included in the Supporting Information (SI).

We have now added the details of the simulation using VASP into the SI.

DFT simulations are carried out with VASP using the PBE exchange-correlation functional and projector augmented wave (PAW) pseudo-potentials. The energy cutoff is 500 eV and the k-point mesh is 18×18×1 for unit-cell calculations. The k-point mash converts to 6×6×1 for the 3×3 supercell.

Another technical issue that needs to be addressed is the type of van der Waals (vdW) correction used in the calculations. In the field of ab initio calculations, there are different types of vdW corrections that can be used, such as nonlocal DF-type vdW correction (originally proposed by Dio et al. published in PRL 92 (2004) 246001), which includes DF, DF2, DF3, optPBE, rVV10, and others, as well as semi-empirical D-type vdW correction, which includes D2, D3, D4, Tkatchenko-Scheffler method, and others. The details of the vdW correction used in the calculations are not provided in the manuscript, which makes it difficult to assess the validity of their results. Therefore, it is important for the authors to provide information on the vdW correction they used and how they validated their results to ensure that their calculations are accurate and reliable.

We agree with the referee that including details about the chosen method for describing the vdW forces is important. This comment echoes comment 2 from Reviewer 2. In general, the vdW forces are described by a simple LJ potential trained on DFT calculations including Tkatchenko-Scheffler van der Waals (TS-vdW) corrections with iterative Hirshfeld partitioning. In particular, we use such method to extract the C6 coefficients of the LJ potential. Initially we have employed VASP for the job. However, due to the error in the Hirshfeld partitioning arising from the plane-wave basis set, we moved to FHI-AIMS, which uses numerical atom-centered orbits (and it is all-electron). FHI-AIMS has then been used throughput.

In order to validate the parameters we have computed the binding energy and inter-layer sliding energy surface (ISES) of bilayer MoS_2. The binding energies are 35.7 meV/A^2 (DFT-TS) and 32.1 meV/A^2 (SNAP-LJ), and the ISES corrugations are 23.3 meV/A^2 (DFT-TS) and 16.3 meV/A^2 (SNAP-LJ).

We have added a note in the Supporting Information (SI) on the results mentioned above and the correct pointer into the main manuscript. The note reads:

The inter-layer interaction is computed through a Lennard-Jones (LJ) potential. The C6 coefficients are extracted from the Tkatchenko-Scheffler van der Waals (TS-vdW) corrections with iterative Hirshfeld partitioning. FHI-AIMS is used with the “tight” version of its numerical atom-centered orbits. The extracted LJ coefficients are listed in Table III. To test the interlayer potential we calculate the inter-layer sliding energy surface (ISES) of bilayer MoS2 using both DFT and the LJ potential, which return us an ISES corrugation of 23.3 meV/˚A^2 and 16.3 meV/˚A^2, respectively.

Additional Questions:
Urgency: High
Significance: High
Novelty: Top 10%
Scholarly Presentation: High
Is the paper likely to interest a substantial number of physical chemists, not just specialists working in the authors' area of research?: Yes

jz-2023-010666.R2

Name: Peer Review Information for "Stiffness and Atomic-Scale Friction in Superlubricant MoS_2 Bilayers"

Second Round of Reviewer Comments

Reviewer: 4

Comments to the Author

The authors have thoroughly revised the manuscript and provided an excellent response. I have no additional question or comment.

Reviewer: 2

Comments to the Author

The authors have now provided an honest answer showing that their simulations do not agree with experimental observations. There are many reasons for such a disagreement. However, one of such prominent reasons is the crude treatment of interlayer interactions. I had asked the authors to put their crude Lennard-Jones-like treatment in the context of more advanced models based on registry index and many-body dispersion (MBD) calculations. The fact that a LJ-like potential can be fitted to yield an arbitrary corrugation does not mean that it is a reliable approach for fully-blown MD simulations of frictional phenomena. The authors ought to add a discussion about high-accuracy calculations of interlayer potentials in the literature (see Refs in my first report) and put their results (wrt disagreement with experiment) in this context. Once this is done, I can recommend publication.

Author's Response to Peer Review Comments:

Dear Prof. Editor,

We thank your office for handling the review of our manuscript, and the reviewers for their reports, which give us the opportunity to improve our manuscript. We are pleased to note that the referees suggest no further review is needed once we adapt the required change.

Below we address the reviewer's comment and indicate the modifications made to the revised manuscript. For convenience, the referees' comments are in black, our reply in blue and the changes made to the manuscript in red. As you will see, we have adapted the suggestion and we believe that the manuscript is now suitable for publication in the Journal of Physical Chemistry Letters.

Thank you for your consideration, Best regards,

Rui Dong, Alessandro Lunghi and Stefano Sanvito

Reviewer: 2

Recommendation: This paper is probably publishable, but major revision is needed; I do not need to see future revisions.

Comments: The authors have now provided an honest answer showing that their simulations do not agree with experimental observations. There are many reasons for such a disagreement. However, one of such prominent reasons is the crude treatment of interlayer interactions. I had asked the authors to put their crude Lennard-Jones-like treatment in the context of more advanced models based on registry index and many-body dispersion (MBD) calculations. The fact that a LJ-like potential can be fitted to yield an arbitrary corrugation does not mean that it is a reliable approach for fully-blown MD simulations of frictional phenomena. The authors ought to add a discussion about high-accuracy calculations of interlayer potentials in the literature (see Refs in my first report) and put their results (wrt disagreement with experiment) in this context. Once this is done, I can recommend publication.

Reply: We thank the reviewer for additional comments and apologize for overlooking this point in the first revised version of our manuscripts.

We have amended the discussion and added the mentioned references into the manuscripts:

A Lennard-Jones (L-J) potential, with C6 coefficients extracted from van der Waals DFT calculations [30] describes the inter-layer interaction. Similar results are obtained for other bilayer polymorphs. It is worth to note more sophisticated inter-layer potential have been developed based on registry index and many-body dispersion.[41-43] In the case of graphene and h-BN, the newly developed potentials accurately predict the ISES corrugation, and have subversively advantage over simple L-J potential. Studies also show that many-body vdW effects may become more prominent for non-equilibrium geometries.[44] In this work, the simple L-J potential returns a bilayer binding energy of 32.1 eV/˚A^2 and a ISES corrugation of 16.3 meV/A^2 for the 2H order. The DFT values are 35.7 eV/˚A^2 and 23.3 meV/˚A^2, respectively. Therefore, a L-J potential should be enough to deliver the physics studied in this work, namely, to identify the role of the materials stiffness on superlubricity.

with the new references:

(41) Gao, W.; Tkatchenko, A. Sliding Mechanisms in Multilayered Hexagonal Boron Nitride and Graphene: The Effects of Directionality, Thickness, and Sliding Constraints. Phys. Rev. Lett., 2015, 114(9), 096101, 1-5. DOI: 10.1103/PhysRevLett.114.096101

(42) Hauseux, P.; Ambrosetti, A.; Bordas, S. P.A; Tkatchenko, A. Colossal Enhancement of Atomic Force Response in van der Waals Materials Arising from Many-Body Electronic Correlations. Phys. Rev. Lett., 2022, 128(10), 106101, 1-6. DOI: 10.1103/PhysRevLett.128.106101

(43) Ouyang, W.; Sofer, R.; Gao, X.; Hermann, J.; Tkatchenko, A.; Kronik, L.; Urbakh, M.; Hod, O. Anisotropic Interlayer Force Field for Transition Metal Dichalcogenides: The Case of Molybdenum Disulfide. J. Chem. Theory Comput., 2021, 17(11), 7237-7245. DOI: 10.1021/acs.jctc.1c0078218

(44) Galante, M.; Tkatchenko, A. Anisotropic van der Waals dispersion forces in polymers: Structural symmetry breaking leads to enhanced conformational search. Phys. Rev. Research, 2023, 5(1), L012028, 1-6. DOI: 10.1103/PhysRevResearch.5.L012028

Furthermore, we have added the following sentence at the end of the discussion on the comparison with experimental data:

….. Note also that the use of a more sophisticated description of the dispersive forces may improve the quantitative description.
